# The Comparison of Iranian Normative Reference Data with Five Countries ‎Across Variables in Eight Rorschach Comprehensive System (CS) Clusters

**Published:** 2016-07

**Authors:** Abufazel Hosseininasab, Mohammadreza Mohammadi, Samira Jouzi, Maryam Esmaeilinasab, Ali Delavar

**Affiliations:** 1 Department of Psychology, Allameh Tabatabaei University, Tehran, Iran.; 2 Psychiatry and Psychology Research Center, Tehran University of Medical Sciences, Tehran, Iran.; 3 Department of Psychology, ‎ Science and Research Branch,‎ Islamic Azad University, Tehran, Iran.; 4 Department of Psychology, Tarbiat Modares University, Tehran, Iran.

**Keywords:** *Comprehensive System*, *Iranian Normative Data*, *Rorschach Test*

## Abstract

**Objective: **This study aimed to provide a normative study documenting how 114 five-seven year-old non-‎patient Iranian children respond to the Rorschach test. We compared this especial sample to ‎international normative reference values for the Comprehensive System (CS).‎

**Method:** One hundred fourteen 5- 7- year-old non-patient Iranian children were recruited from public ‎schools. Using five child and adolescent samples from five countries, we compared Iranian ‎Normative Reference Data- based on reference means and standard deviations for each sample.‎

**Results:** Findings revealed that how the scores in each sample were distributed and how the samples were ‎compared across variables in eight Rorschach Comprehensive System (CS) clusters. We reported ‎all descriptive statistics such as reference mean and standard deviation for all variables.‎

**Conclusion**: Iranian clinicians could rely on country specific or “local norms” when assessing children. We ‎discourage Iranian clinicians to use many CS scores to make nomothetic, score-based inferences ‎about psychopathology in children and adolescents.‎

Establishing accurate normative data for the Rorschach (1) Comprehensive System method (CS; ‎‎^2^) is crucial to its use in clinical practice. As with other tests, Rorschach interpretation rests on (a) ‎quantitative, nomothetic normative comparisons, and (b) qualitative idiographic, individualized ‎inferences. Thus, evaluating deviations from normative expectations is a central component in ‎quantitative interpretation.‎

However, the adequacy of the CS adult and child reference values (2) has been discussed and ‎debated in the literature over the past decade, both with respect to samples from the U.S. (e.g., 3; ‎‎^4^; ^5^) and other countries (e.g., 6; 7; 8; 9; 10). The available CS norms for children and ‎adolescents were first published in 1982 (11). At the time, the authors questioned how ‎representative their samples were, cautioning users that as a result of likely self- and parent-‎selection biases they were probably too healthy and well-functioning to generalize to typical ‎participants. Therefore, it is of prime importance to identify what more recently collected samples ‎look like when plotted on the Adult Composite International Norms.‎

A study that sparked concern about the standard CS reference values was Shaffer et al.’s (1999) ‎sample of 123 adults from Fresno, California (4). These participants were tested by graduate ‎students, that Weiner (2001) questioned as a suitable level of training and experience to serve as ‎a reference sample (12). Nonetheless, because both the Fresno sample and the traditional CS ‎norms were obtained from non-patients in the U.S., any disparities between them were notable. ‎In particular, Shaffer et al. Reported many shorter and more simplistic records than the existing ‎CS norms. For instance, their sample had a mean R = 20.8 (versus 23.5 in the CS norms) and a ‎mean Lambda = 1.22 (vs. 0.58), with 41% of their sample classified as having an avoidant style ‎‎ (i.e., Lambda > 0.99; vs. 14%).‎

Wood et al. (2001a, 2001b) showed that a number of non-patient samples from the literature ‎produced notably different mean values from the CS norms (CS 600), with effect sizes ranging ‎from small to very large. 

Form quality (FQ)-related (i.e., XQ%, X–%) and color-related variables ‎‎(i.e., Afr, FC, WSumC), as well as popular (P), whole, realistic human content (Pure H), diffuse ‎shading (Y), and reflection (Fr, rF) responses were the most problematic variables (5). Other ‎empirical evidence also showed that the distributions for form quality (FQ) and number of ‎responses (R) might diverge from the CS normative expectations in non-patient samples (11). ‎

Meyer et al. (2007) presented descriptive data from 4,704 Rorschach records from non-patient ‎samples from Argentina, Australia, Belgium, Brazil, Denmark, Finland, France, Greece, Israel, ‎Israel, Italy, Japan, Peru, Portugal, Romania, Spain, the Netherlands, and the United States (13). ‎The mean age of the entire, combined sample was 36.65 (SD = 11.71). Years of education, ‎gender, and race were not reported. Analyses of these international data revealed that both the ‎CS 450 collected by Exner and Erdberg (2005) and the CS 600 collected by Exner (2001) ‎diverged from most of the other samples for a large proportion of the variables (14) (15). ‎Applying CS 600 interpretive routines to all these samples may result in pathologized ‎interpretation of these non-patients. Viglione and Meyer (2008) summarized the recurring main ‎differences between the CS 600 and other samples and reported that other samples frequently ‎produced more unusual location responses, inferior form quality, fewer elaborated, positive ‎human representations, less color, and fewer texture responses (16). To provide the Iranian ‎Rorschach users with more representative normative benchmarks and to reduce the risk of overly ‎pathological interpretations, we presented an Iranian normative reference sample. In this study, ‎we extended the previous analyses in several ways. First, we reported data collected for 5 to 7 ‎year- old Iranian children. Second, we utilized international normative reference values for the ‎CS for children and adolescents (2). Third, and most important, we focused on the extent to ‎which international normative reference values for the CS correspond to Iranian sample.‎‎

## Materials and Method

***Participants***

The sample for this study consisted of 114 non-patient Iranian children aged 5 -7 who were ‎recruited from public schools. Recruitment began with identifying an area of Tehran that has ‎numerous schools and is demographically representative of its population. Within this area, 15 ‎public schools and five kindergartens were randomly selected. The principals of the schools sent ‎a letter to the parents in which they described the purpose of the study–to collect normative ‎reference data on a psychological test–and asked them to sign and return the letter if they agreed ‎to have their children participate in the study. Before sending the letters, the principals reviewed ‎their school records and removed from the list any students who had been identified as having ‎psychological problems. Similarly, the parents were asked not to sign and return the letter of ‎agreement if their child had been diagnosed with or treated for any psychological disorder within ‎the last two years or had undergone psychological testing within the past year. Approximately ‎‎90% of the parents signed the letter. Children’s participation in the study was voluntary and ‎required their consent as well as their parents’ approval. The final sample of 114 participant's ‎included 49 (43%) boys and 65 (57%) girls.‎

***Procedures***

The Rorschach data were collected by 15 examiners; all of whom were graduate students of the ‎Allameh Tabatabaei University, who have completed an assessment course that included ‎instruction in the administration, coding and interpretation of the Rorschach; and they were ‎currently enrolled in a two-year Rorschach research practicum co-mentored by the second author. ‎All of the examinations were conducted in Farsi in the counseling rooms of the students’ school, ‎following a brief warm-up and the completion of a short semi-structured interview. The ‎Rorschach administration was conducted according to Exner’s (2003) Comprehensive System ‎instructions, including his procedures for obtaining at least 14 responses and avoiding excessively ‎long protocols (2).‎ 

***Samples and Procedures for Comparison***

In 2007, a supplemental issue of the Journal of Personality Assessment was devoted to ‎international normative data for Rorschach Comprehensive System. In this special issue, a ‎number of investigators collected 39 samples from 17 countries (18). These international ‎reference data included samples of children and adolescents from Denmark (19), Italy (20), ‎Japan (21) Portugal (22) and United States (CS; 2). Subsequent publications have presented ‎Rorschach reference data on samples of non-patient children in Brazil (23) and adolescents in ‎Israel (24). The samples differed in their quality (e.g., examiner training, scoring reliability, and ‎checks on administration quality); however, motivated and trained individuals seeking to ‎advance the database of Rorschach assessment collected all the data. ‎

To compare normative reference values for the CS, we used the Four Children Samples published ‎in special issue of the Journal of Personality Assessment in 2007 (JPA, 89, Suppl.1). Countries ‎that reported normative data were Italy (20), Japan (21), Portugal (22), Brazil (23) and traditional ‎CS reference data (2).‎

## Results

Table 1 demonstrates descriptive statistics for each Rorschach variable. Using the descriptive ‎data in Table 1, we reported all descriptive statistics such as reference mean and standard ‎deviation for all variables.

**Table1:** Descriptive Statistics for Non-Patient Iranian Children Aged 5-7 (N = 114) a

**5-6-7 years (N = 114)**									
**Variable**	**Mean**	**SD**	**Min**	**Max**	**Freq**	**Median**	**Mode**	**SK**	**KU**
R	21.60	5.33	14.00	36.00	114	20	17	0.71	-0.09
W	7.12	4.08	1.00	23.00	114	6	4	1.02	1.16
D	11.25	5.88	0.00	25.00	112	11	15	0.15	-0.61
Dd	3.18	2.41	0.00	18.00	103	3	2	2.22	11.32
S	1.09	1.31	0.00	6.00	64	1	0	1.96	4.50
DQ+	2.18	2.48	0.00	10.00	77	1	0	1.23	0.72
DQo	16.37	5.84	5.00	33.00	114	15.5	12	0.67	0.11
DQv+/	0.10	0.35	0.00	2.00	9	0	0	3.92	15.89
DQv	2.94	2.32	0.00	11.00	98	3	3	0.90	0.78
FQx+	0.20	0.92	0.00	8.00	9	0	0	6.44	48.12
FQxo	7.28	2.91	2.00	17.00	114	7	6	0.57	0.53
FQxu	7.09	3.43	1.00	19.00	114	6	6	0.75	0.77
FQx-	5.95	3.32	0.00	18.00	113	6	4	0.70	0.65
FQxNone	1.06	1.72	0.00	7.00	46	0	0	1.81	2.54
MQ+	0.14	0.82	0.00	8.00	6	0	0	8.15	73.72
MQo	0.49	0.84	0.00	4.00	37	0	0	1.91	3.49
MQu	0.39	0.67	0.00	3.00	35	0	0	1.80	3.16
MQ-	0.31	0.70	0.00	4.00	25	0	0	2.81	8.99
MQNone	0.05	0.26	0.00	2.00	5	0	0	5.47	32.44
SQual-	0.26	0.56	0.00	3.00	25	0	0	2.66	8.64
M	1.38	1.96	0.00	13.00	67	1	0	2.70	10.87
FM	2.36	2.21	0.00	14.00	90	2	0	1.73	5.78
m	1.43	1.58	0.00	6.00	71	1	0	1.17	0.76
FC	1.08	1.31	0.00	6.00	66	1	0	1.50	2.19
CF	0.56	0.74	0.00	3.00	49	0	0	1.17	0.80
C	1.25	1.56	0.00	7.00	66	1	0	1.49	1.94
Cn	0.06	0.35	0.00	3.00	4	0	0	6.67	47.57
SumC	2.96	2.12	0.00	10.00	103	2	2	0.85	0.46
WSumC	3.02	2.42	0.00	12.00	103	2.5	2.5	1.20	1.57
FC’	0.73	1.04	0.00	5.00	51	0	0	1.65	2.83
C’F	0.27	0.66	0.00	3.00	21	0	0	2.87	8.29
C’	0.40	1.17	0.00	11.00	28	0	0	6.86	59.54
FT	0.15	0.45	0.00	2.00	14	0	0	2.96	8.28
TF	0.09	0.32	0.00	2.00	10	0	0	3.52	12.91
T	0.08	0.41	0.00	3.00	6	0	0	5.29	29.75
FV	0.10	0.35	0.00	2.00	9	0	0	3.92	15.89
VF	0.00	0.00	0.00	0.00	0	0	0		
V	0.00	0.00	0.00	0.00	0	0	0		
FY	0.02	0.13	0.00	1.00	2	0	0	7.44	54.42
YF	0.06	0.27	0.00	2.00	6	0	0	4.91	26.13
Y	0.06	0.27	0.00	2.00	6	0	0	4.91	26.13
Fr	0.01	0.09	0.00	1.00	1	0	0	10.67	114.00
rF	0.00	0.00	0.00	0.00	0	0	0		
Sum C’	1.41	1.97	0.00	15.00	70	1	0	3.43	19.26
SumT	0.34	0.64	0.00	3.00	29	0	0	1.89	2.96
SumV	0.09	0.35	0.00	2.00	9	0	0	3.92	15.89
SumY	0.14	0.45	0.00	3.00	12	0	0	3.89	17.13
Sum Shading	1.99	2.11	0.00	15.00	86	1.5	1	2.50	11.71
Fr+rF	0.01	0.09	0.00	1.00	1	0	0	10.67	114.00
FD	0.44	0.83	0.00	5.00	34	0	0	2.47	8.07
F	12.50	5.55	0.00	30.00	113	12	15	0.47	0.15
PAIR	6.11	3.55	0.00	16.00	109	6	7	0.31	-0.36
3r+(2)/r	0.26	0.14	0.00	0.63	110	0.27	0.14	0.16	-0.67
Lambda	2.24	2.68	0.00	15.00	113	1.33	1	2.66	7.89
PureF%	0.56	0.20	0.00	0.94	113	0.57	0.50	-0.24	-0.38
FM+m	3.77	2.93	0.00	19.00	103	3	1	1.50	5.21
EA	4.39	3.14	0.00	19.50	108	3.5	2.50	1.37	3.80
es	5.65	3.54	0.00	19.00	108	5	6	0.71	0.88
D Score	-0.32	1.28	-5.00	5.00	114	0	0	-0.18	4.27
AdjD	-0.09	1.12	-4.00	5.00	114	0	0	0.34	5.69
Active (a)	3.39	3.23	0.00	18.00	97	3	3	1.76	4.45
Passive (p)	1.72	1.91	0.00	8.00	76	1	0	1.21	0.83
Ma	0.98	1.74	0.00	12.00	53	0	0	3.29	14.74
Mp	0.39	0.69	0.00	3.00	33	0	0	1.80	2.78
Intellect	1.12	1.80	0.00	12.00	55	0	0	2.95	12.57
Zf	7.54	4.23	1.00	23.00	114	7	4	1.29	2.06
Zd	-1.89	4.81	-24.00	8.50	114	-1.75	-0.5	-0.76	3.23
Blends	1.79	2.09	0.00	9.00	74	1	0	1.25	0.75
Blends/R	0.09	0.11	0.00	0.52	74	0.05	0	1.48	1.93
Col-Shd-Blends	0.47	0.84	0.00	4.00	36	0	0	2.06	4.12
Afr	0.49	0.16	0.12	1.18	114	0.50	0.50	0.66	1.57
Populars	2.84	1.54	0.00	7.00	110	3	2	0.38	-0.44
XA%	0.67	0.14	0.31	1.00	114	0.67	0.64	-0.21	-0.34
WDA%	0.69	0.14	0.21	1.00	114	0.70	0.64	-0.40	0.10
X+%	0.35	0.14	0.08	0.78	114	0.35	0.25	0.29	-0.03
X-%	0.26	0.13	0.00	0.63	113	0.27	0.33	0.30	-0.32
Xu%	0.32	0.13	0.04	0.69	114	0.32	0.25	0.11	-0.05
Isolate/R	0.18	0.17	0.00	0.76	96	0.14	0	1.19	1.31
H	1.46	1.63	0.00	9.00	80	1	1	1.86	4.49
(H)	0.96	1.26	0.00	6.00	59	1	0	1.68	3.16
HD	1.21	1.72	0.00	10.00	64	1	0	2.31	6.77
(HD)	0.23	0.73	0.00	5.00	16	0	0	4.49	23.15
Hx	0.32	0.71	0.00	4.00	24	0	0	2.66	7.94
All H Cont	3.87	2.73	0.00	14.00	108	4	4	1.25	2.34
A	8.57	4.10	0.00	23.00	111	8	8	0.54	0.73
(A)	0.34	0.68	0.00	4.00	29	0	0	2.57	8.17
Ad	1.58	2.42	0.00	12.00	57	0.50	0	2.02	4.30
(Ad)	0.02	0.16	0.00	1.00	3	0	0	5.99	34.57
An	0.50	0.90	0.00	4.00	34	0	0	1.97	3.59
Art	0.61	1.02	0.00	5.00	43	0	0	2.28	6.06
Ay	0.02	0.13	0.00	1.00	2	0	0	7.44	54.42
BI	0.14	0.51	0.00	3.00	10	0	0	3.66	13.210
Bt	1.16	1.40	0.00	6.00	63	1	0	1.19	0.79
Cg	1.24	1.39	0.00	6.00	72	1	0	1.37	1.79
CI	0.03	0.22	0.00	2.00	3	0	0	7.15	54.66
Ex	0.04	0.20	0.00	1.00	5	0	0	4.51	18.70
Food	0.37	0.75	0.00	4.00	30	0	0	2.60	8.21
Fi	0.64	1.10	0.00	6.00	44	0	0	2.41	7.00
Ge	0.01	0.09	0.00	1.00	1	0	0	10.67	114.00
Hh	0.61	0.90	0.00	4.00	45	0	0	1.59	2.04
Ls	0.69	1.09	0.00	5.00	46	0	0	1.90	3.34
Na	0.85	1.24	0.00	6.00	55	0	0	2.05	4.87
Sc	1.07	1.22	0.00	6.00	69	1	0	1.48	2.62
Sx	0.01	0.09	0.00	1.00	1	0	0	10.67	114.00
Xy	0.02	0.20	0.00	2.00	2	0	0	8.53	76.01
Idiographic	0.71	1.13	0.00	5.00	44	0	0	1.74	2.39
An+Xy	0.51	0.91	0.00	4.00	34	0	0	1.91	3.30
DV	0.53	0.99	0.00	6.00	38	0	0	2.73	9.43
INCOM	1.14	1.42	0.00	9.00	67	1	0	2.12	7.46
DR	0.24	0.69	0.00	4.00	17	0	0	3.42	12.37
FABCOM	0.28	0.63	0.00	4.00	25	0	0	3.13	12.77
DV2	0.03	0.18	0.00	1.00	4	0	0	5.12	24.65
INC2	0.23	0.62	0.00	4.00	18	0	0	3.26	12.64
DR2	0.03	0.18	0.00	1.00	4	0	0	5.12	24.65
FAB2	0.02	0.13	0.00	1.00	2	0	0	7.44	54.42
ALOG	0.71	1.12	0.00	6.00	49	0	0	2.37	6.78
CONTAM	0.06	0.27	0.00	2.00	6	0	0	4.91	26.13
Sum 6Sp Sc	3.27	2.52	0.00	15.00	103	3	2	1.64	4.61
Lvl 2 Sp Sc	0.28	0.63	0.00	3.00	22	0	0	2.27	4.48
WSum6	9.74	8.01	0.00	39.00	104	8	6	1.23	1.41
AB	0.24	0.73	0.00	6.00	18	0	0	5.01	33.53
AG	0.23	0.67	0.00	4.00	17	0	0	3.47	13.21
COP	0.20	0.61	0.00	3.00	15	0	0	3.62	13.51
CP	0.02	0.13	0.00	1.00	2	0	0	7.44	54.42
Good HR	2.20	1.72	0.00	10.00	95	2	2	1.16	2.85
Poor HR	2.21	2.30	0.00	10.00	89	1	1	1.57	2.56
MOR	0.72	1.45	0.00	3.00	40	0	0	3.21	12.69
PER	0.47	0.88	0.00	4.00	36	0	0	2.42	6.19
PSV	0.42	0.79	0.00	3.00	32	0	0	1.93	3.03
PTI Total	1.28	1.13	0.00	4.00	76	1	0	0.42	-0.66
DEPI Total	2.97	1.35	0.00	6.00	106	3	3	-0.41	0.23
CDI Total	3.29	1.14	0.00	5.00	112	3	4	-0.66	0.17
SCon Total	3.17	2.41	0.00	7.00	83	4	0	-0.09	-1.39
HVI Total	0.39	0.79	0.00	4.00	28	0	0	2.18	4.70
OBS Total (1-5)	0.25	0.48	0.00	2.00	25	0	0	1.86	2.72
EII-3	0.42	0.63	0.00	2.50	62	0.10	0	1.79	2.65
HRV	-0.18	2.81	-9	6.00	97	0	1	-0.776	1.66
W+D	18.33	4.92	10.00	34.00	114	17.5	14	0.73	0.16
EBPer	0.49	1.34	0.00	6.50	16	0	0	3.00	8.86

a. The Comprehensive System codes that correspond to the variable names in the first column are as follows: Number of Responses (R); Lambda (L); Human Movement (M); Weighted Sum of Color (WSumC); Experience Actual(EA); Animal Movement (FM); Inanimate Movement (m); Nonhuman Movement (FM + m); Diffuse Shading (SumY); Texture (SumT); Vista (SumV); Achromatic Color (SumC’); Sum of Shading (SumShd); Experienced Stimulation (es); Difference Score (D Score); Adjusted Difference Scale (AdjD); Coping Style (Erlebnistypus, EB); White Space (S); Color Projection (CP); Form-Color Ratio (CF+C: FC); Pure Color (Pure C); Affective Ratio (Afr); Complexity Ratio (Blends:R); Constriction Ratio (SumC’:WSumC); Aggressive Movement (AG); Cooperative Movement (COP); Food (Fd); Personal (PER); Active:Passive Ratio (a:p or Ma:Mp); Whole, Realistic Humans (Pure H or H: (H) + Hd + (Hd)); Interpersonal Interest (SumH H+ (H)+Hd+ (Hd)); Good and Poor Human Representations (GHR and PHR); Morbid (MOR); Anatomy and X-ray (An + Xy); Reflections (Fr + rF); Form Dimension (FD); Synthesized Response (DQ+); Vague Response (DQv); Perseveration (PSV); Organizational Frequency (Zf); Processing Efficiency (Zd); Aspiration Ratio (W:M); Economy Index (W:D:Dd); Form Quality Scores: Conventional (X+%), Appropriate (WDA%), Unusual (Xu%), Distorted (X%); White Space Distortion (S); Popular (P); Human Movement With Distorted Form (M); Human Movement, Formless (Mnone); Critical Special Scores (Sum6 or WSum6); and Critical Special Scores, Severe (Level 2)

**Table2:** Rorschach Variable Frequencies for 114 Non-patient Iranian Children and Four Country

	**Iran 5-7** **N=114**		**Italy 5-7** **N=75**		**Japan 5-6** **N=24**		**Portugal 6-7** **N=155**		**Brazil 7** **N=50**	
**Styles**	**Freq**	**%**	**Freq**	**%**	**Freq**	**%**	**Freq**	**%**	**Freq**	**%**
Introversive	8	7	7	9	0	0	7	5	0	0
Pervasive Introversive	5	4	4	5	0	0	4	3	0	0
Ambitent	13	11	13	17	0	0	6	4	2	4
Extratensive	20	18	4	5	0	0	12	8	7	14
Pervasive Extratensive	10	9	2	3	0	0	9	6	3	6
Avoidant	73	64	51	68	24	100	70	45	41	82
**D-Scores**										
D Score > 0	12	11	9	12	1	4	27	17	12	24
D Score = 0	67	59	37	49	21	88	101	65	9	18
D Score < 0	35	31	29	39	2	8	27	17	29	58
D score < −1	15	13	15	20	0	0	9	6	22	44
Adj D Score > 0	15	13	9	12	1	4	27	17	13	26
Adj D Score = 0	75	66	41	55	21	88	105	68	11	22
Adj D Score < 0	24	21	25	33	2	8	23	15	26	52
Adj D score < −1	10	9	9	12	0	0	6	4	20	40
Zd > +3.0 (Overincorp)	10	9	19	25	3	13	23	15	7	14
Zd < −3.0 (Underincorp)	40	35	18	24	2	8	47	30	7	14
**Form Quality**										
XA > 0.89	6	5	4	5	0	0	1	1	0	0
XA < 0.70	66	58	54	72	24	100	95	61	14	28
WDA% < 0.85	95	83	68	91	24	100	139	90	41	82
WDA% < 0.75	69	61	52	69	24	100	107	69	34	68
X+% < 0.55	105	92	73	97	24	100	139	90	46	92
Xu% > 0.20	94	83	59	79	2	8	113	73	30	60
X−% > 0.20	75	66	67	89	24	100	133	86	44	88
X−% > 0.30	41	36	51	68	24	100	85	55	32	64
**FC:CF+C Ratio**										
FC > (CF+C)+2	6	5	11	15	0	0	6	4	0	0
FC > (CF+C)+1	16	14	18	24	1	4	17	11	3	6
(CF+C) > FC+1	40	35	10	13	3	13	48	31	16	32
(CF+C) > FC+2	19	17	5	7	0	0	26	17	8	16
**S-Constellation Positive**										
HVI Positive	0	0	10	13	4	17	17	11	0	0
OBS Positive	0	0	0	0	0	0	0	0	0	0
PTI = 5	0	0	2	3	0	0	0	0	0	0
PTI = 4	4	4	12	16	0	0	0	0	2	4
PTI = 3	11	10	19	25	23	96	43	28	16	32
DEPI = 7	0	0	0	0	0	0	0	0	0	0
DEPI = 6	3	3	10	13	0	0	10	6	0	0
DEPI =5	8	7	22	29	0	0	31	20	6	12
CDI = 5	13	11	15	20	5	21	16	10	3	6
**Miscellaneous Variables**										
R < 17	20	18	35	47	12	50	21	14	36	72
R > 27	18	16	15	20	0	0	38	25	0	0
DQv > 2	59	52	17	23	3	13	61	39	6	12
S > 2	12	11	32	43	2	8	54	35	7	14
Sum T = 0	86	75	64	85	24	100	133	86	42	84
SumT > 1	9	8	3	4	0	0	7	5	3	6
3r+(2)/R < 0.33	75	66	50	67	23	96	110	71	36	72
3r+(2)/R > 0.44	13	11	3	4	0	0	18	12	36	72
Fr+rF > 0	1	1	11	15	0	0	3	2	3	6
Pure C > 0	66	58	4	5	4	17	54	35	17	34
Pure C > 1	34	30	20	27	1	4	26	17	8	16
Afr < 0.40	27	24	41	55	4	17	37	24	13	26
Afr < 0.50	54	48	27	36	8	33	64	41	18	36
(FM+m) < Sum Shading	21	18	27	36	0	0	41	26	15	30
(2AB+ART+AY) > 5	2	2	1	1	0	0	2	1	0	0
Populars < 4	78	68	57	76	19	79	98	63	37	74
Populars > 7	0	0	0	0	0	0	5	3	0	0
COP = 0	99	87	64	85	21	88	139	90	47	94
COP > 2	4	4	1	1	0	0	2	1	0	0
AG = 0	97	85	67	89	21	88	121	78	44	88
AG > 2	3	3	0	0	0	0	3	2	0	0
MOR > 2	10	9	2	3	0	0	16	10	0	0
Level 2 Sp.Sc. > 0	22	19	18	24	1	4	21	14	3	6
GHR > PHR	50	44	24	32	2	8	55	35	15	30
Pure H < 2	76	67	59	79	19	79	89	57	38	76
Pure H = 0	34	30	43	57	14	58	43	28	23	46
p > a+1	18	16	4	5	1	4	21	14	5	10
Mp > Ma	19	17	10	13	3	18	35	23	6	12

For instance, Table 1 shows that R has M = 21.60 and SD = 5.33. ‎Reference mean and standard deviation allow one to determine quickly how far a person or a ‎sample is from the expected norms and to see how typical or atypical values are for the person or ‎sample compared to the norms.‎

Table 2 displays the frequency and percentage of Rorschach variables of the 114 non-patient ‎Iranian children and international normative reference values for the CS. To facilitate cross-‎national comparisons, we presented miscellaneous variables for all samples. This table presents ‎country specific distribution for the important scores, which were not listed in Table 1 (i.e., ‎Styles, D-Scores, Form Quality, S-Constellation Positive and so on). Table 2 shows how the ‎scores are distributed within each country. Given that positive and negative deviations from the ‎mean cancel out, the most salient information in this table is the dispersion of scores.‎

## Discussion

Based on the seven clusters proposed by Exner (2), we briefly present the key data concerning ‎normative findings to emphasize how Iranian children respond to the Rorschach: ‎

***Information Processing***

As displayed in Table 2, with respect to location, predominance of D as opposed to W is clear in ‎‎5-7- year-old Iranian children (11.25 vs. 7.12). This ratio is similar to the results from other ‎countries (Brazil: 7.70 vs. 5.18; Italy: 8.01 vs. 7.01; Japan: 7.17 vs. 6.88; and Portugal: 10.84 vs. ‎‎9.17) except for data from the United States (8.04 vs. 10.36). For developmental quality, ‎complicated responses (DQ+) occurred with lower frequency than simple responses (DQo; 2.18 ‎vs. 16.37). This pattern is in parallel with previous studies (Brazil: 1.90 vs. 13.04; Italy: 3.52 vs. ‎‎15.95; Japan: 1.83 vs. 14.54; and Portugal: 4.01 vs. 16.97; US: 5.46 vs. 11.06). The mean number ‎of the responses that are indicative of organizational activity was as expected (Iran: 7.54; Brazil: ‎‎6.64; Portugal: 11.26; Japan: 7.04; Italy: 8.99). The percentage of under-corporative children was ‎equal to 35% (compared with Brazil: 14%; Italy: 22%; Japan: 8%; and Portugal: 30%). ‎

***Mediation***

Due to the simple nature of information processing in childhood, it is not surprising that mean ‎lambda in 5-7 –year-old Iranian children was higher (2.24) than those expected from adults. ‎However, lambda value of Iranian children was lower than the value in most of the other ‎countries (Brazil: 4.13; Italy: 3.02; Japan: 8.47; Portugal: 3. 76) except US (1.46). The other ‎factor necessary to discuss is the form quality. XA% was equal to 0.67. Similar results are ‎presented in other studies (Brazil: 61%; Portugal: 65%; Japan: 35%; Italy: 61%; America: 91%). ‎

***Ideation***

In this cluster, there was not any notable data except for lack of M (1.38 vs. compared to Brazil: ‎‎0.48; Portugal: 1.56; Japan: 0.71; Italy: 1.33; America: 2.23) and FM (2.36 vs. Brazil: 1.52; ‎Portugal: 1.87; Japan: 0.96; Italy: 2.27; America: 5.15).‎

***Controls and Stress Tolerance***

According to the high lambda, there was an exception that median of other determents were low. ‎This was also noticeable in FM and also C, shading response and T. In fact, the mean of C was ‎equal to 2.96 in the 5-7 year- old sample (in comparative with Brazil: 1.88; Portugal: 2.88; Japan: ‎‎1.13; Italy: 2.95; America: 5.37). The mean of Sum T and Sum Y were equal to 0.34 and 0.14, ‎respectively (compared to Brazil: 0.24 and 0.72; Portugal: 0.19 and 0.24; Japan 0.0 and 0.0; Italy: ‎‎0.25 and 0.43; America: 0.86 and 0.38). The mean of Sum C’ in 5-7- year- old sample was ‎relatively high (1.41 compared to Brazil: 0.48; Portugal: 1.20; Japan: 0.33; Italy: 1.59; America: ‎‎0.82). Another important data noticeable in Iranian normative data was Afr, whose value was ‎equal to 0.49 (compared to Brazil: 0.56; Portugal: 0.56; Japan: 0.61; Italy: 0.48; America: 0.85).‎

***Affect***

Iranian children provided a small number of responses with color determinants, and this may be ‎due to the high Lambda values cutting through other samples. Inspection of the Afr value gave ‎rise to a new fact when explaining the reason for this decrease. Afr mean values was 0.49 in the ‎Iranian children aged 5 -7. The last point warranting our attention was the fact that CF mean ‎values were always lower than FC ones, with the exception for the 5-7- year- old group, whose ‎values were 0.56 and 1.08, respectively.‎

***Self-perception***

The Egocentricity index was rather low: 0.26 (compared to Brazil: 0.21; Portugal: 0.24; Japan: ‎‎0.09; Italy: 0.23; America: 0.67). This was mainly due to the rare frequency of reflection ‎responses (Fr and rF). Fr and rF mean value obtained for I Iranian children aged 5 to 7 was 0.01 (compared to Brazil: 0.06; Portugal: 0.02; Japan: 0.0; Italy: 0.03; America: 0.32). On the ‎contrary, An and MOR values were consistently high in all groups (compared to Brazil: 0.50 and ‎‎0.72; Portugal: 0.77 and 0.89; Japan: 0.58 and 0.21; Italy: 0.47 and 0.40; America: 0.13 and ‎‎0.83). ‎

***Interpersonal Perception*** ***‎***

Two or three aspects should be emphasized considering some of the variables. The H and Hd ‎values, which are close to each other, vary between 1.46 and 1.21. Because of the ‎aforementioned small values of movement responses, cooperative and aggressive codes were ‎reduced. On the other hand, the Coping Deficit index reached high frequencies in our samples. ‎The percentage of CDI ≥ 4 values of Iranian children aged 5- 7 was 11% (compared to Brazil: ‎‎6%; Portugal: 10%; Japan: 21%; Italy: 20%; America: 1%). ‎

As demonstrated in table 2, responding styles, some key variables, percentage, ratios, and ‎derivations for each of the countries have been presented. In all countries except for the U.S., the ‎domain style was avoidant. All responding styles were similar in Iran, Italy, Portugal and Brazil, ‎but avoidant style was dominated in Japan’s sample. In American normative data, all children ‎had an extroversive style. One important issue about Iranian normative data was that extroversive ‎style was dominated in this sample (18% compared to Brazil: 14%; Portugal: 8%; Japan: 0%; ‎Italy: 5%). Extroversive style was very high in American normative data (56%). There were no ‎noticeable data on D score. In Iranian normative data, ZD<3.0 was hardly high (35% compared ‎to Brazil: 14%; Portugal: 30%; Japan: 8%; Italy: 24%; America: 28%). According to FQ table, ‎XA %> 0.89 was high in Iranian sample. Japan and the U.S. had unusual values. Ratio of ‎FC: CF+C was similar in each of the counters. DQv>2 was relatively high in Iranian normative ‎data (52% compared to Brazil: 12%; Portugal: 39%; Japan: 13%; Italy: 23%; America: 2%). T>1 ‎were similar in all the sample (Iran: 8%, compared with Brazil: 6%; Portugal: 5%; Japan: 0%; ‎Italy: 4%; America: 1%). 3r+ (2)/R < 0.33 in Iranian sample was low. In addition, Pure C>0 and ‎Pure C>1 were high in Iranian normative data (58% and 30%, respectively). Iranian normative ‎data were high in Afr<0.50 (48%). (2AB+ART+AY) > 5 was the highest in Iranian normative ‎data (2%). ‎

## Limitations

The results of this study should be interpreted with caution. These reference data were collected in the city of Tehran and might not generalize to a nonurban population.

## Conclusion

Considering the goal of identifying normative reference values that transcend countries, cultures, ‎languages, recruitment strategies, types of normative target populations, examiner training, and ‎age, the data contained in this study present small different values for the CS in each of the ‎mentioned countries. Although the findings in Meyer, Erdberg & Mihura Supplement (2007), ‎strengthen our ability to use an international normative reference standard for the Rorschach with ‎adults, the data in this study challenge our ability to do so for children and adolescents (25).‎

In agreement with the notation of Meyer and Viglione (2008) that indicated child reference data ‎are unstable, and cautioned clinicians about making inferences on the topic of psychopathology ‎in children from CS data and given the findings of this study, we take this caution further (16). ‎We do not understand the cultural, societal, examiner, and/or administration and scoring factors ‎that are responsible for the erratic results seen with children and adolescents.‎

Finally, it may seem that clinicians could rely on country specific or “local norms” when assessing ‎children. The findings in this study and Meyer, Erdberg & Mihura (2007) leave us concerned that ‎normative information collected by one group in a particular locale may not generalize to the ‎types of data obtained by all clinicians working in that locale (25).‎

## Appendix

**Glossary of Comprehensive System Scores**

A: Content code for whole, real animal

‎(A): Content code for whole, mythical animal‎

a: (active) Superscript for movement determinants denoting a higher level of behavioral output than p (passive)‎

AB: Special Score for coding the use of symbolic representation

Ad: Content code for part or detail of a real animal

‎(Ad): Content code for a part or detail of a mythological animal

AdjD: Adjusted D, calculated by EA – Adj es

Afr Affective Ratio: calculated by responses to Cards VIII+IX+X divided by responses to Cards I+II+III+IV+V+VI+VII

AG: Special Score for coding the use of movement that is aggressive in nature

ALOG: Autistic Logic, Special Score for coding the use of strained reasoning ‎

An: Content code for reference to internal anatomical concepts

An+Xy: Content code for reference to anatomical concepts and X-ray (Xy)‎

Art: Content code for reference to artistic concepts

Ay: Content code for the use of anthropological concepts of a historical or cultural nature

Bl: Content code for the use of blood, whether animal or human

Blends: A response that contains two or more determinants; they are separated by a period

Blends/R: The number of Blends divided by R.‎

Bt: Content code for the use of botanical concepts

C: A determinant reflecting the use of only color in generating the response

C’: A determinant reflecting the use of only achromatic color in generating the response

C’F: A determinant reflecting the emphasis of achromatic color over form in generating the response

CDI Total: The total of all variables that constitute the Coping Deficit Index

CF: A determinant reflecting the emphasis of color over form in generating the response

Cg: Content code for the use of clothing

Cl: Content code for reference to a cloud

Cn: Color naming; a determinant reflecting the use of color by name as the response

Cognitive Special Scores: Special Scores reflecting cognitive slippage (DV, INC, DR, FAB, ALOG, CONTAM)‎

Col-Shd Blends: The total number of Blends combining chromatic and achromatic or shading determinants or both.‎

CONTAM: Contamination, a Special Score reflecting the merging or blending or both of two contents within one blot area

Content: The category in which the response is located

COP: Cooperative Movement, a Special Score reflecting the use of movement (M, FM and m) that is positive or collaborative

CP: Color Projection, a Special Score reflecting the attribution of color to an achromatic portion of the blot

D: Location code indicating a response involving a major detail, one identified in the Card’s location table

D Score: Calculated by EA – es

Dd: Location code indicating response uses an unusual part of the blot, one that may be identified in the Card’s location table

Determinant: A code that reflects how the stimulus field was translated

Developmental Quality: A code reflecting the nature of processing used to produce a response

DQo: A developmental quality code reflecting a single object with specific form demand

DQ+: A developmental quality code reflecting a synthesized answer, one that involves two or more objects, at least one of which has ‎form demand, that are seen in a relationship with one another

DQv: A developmental quality code reflecting a single object with no specific form demand

DQv/+: A developmental quality code reflecting a vaguely synthesized answer, one that involves two or more objects, neither of ‎which has form demand, that are seen in a relationship with one another

DR: Deviant Response, Level one, a Special Score reflecting the use of an inappropriate phrase or circumstantiality

DR2: Deviant response, Level two, a Special Score reflecting the use of an inappropriate phrase or circumstantiality that is bizarre or ‎outside the bounds of reality

DV: Deviant Verbalization, Level one, a Special Score reflecting the use of a neologism, an individualized meaning, or redundancy

DV2: Deviant Verbalization, Level two, a Special Score reflecting the use of a neologism, an individualized meaning, or redundancy

EA: Experience Actual, the addition of Sum M and WSumC.‎

Egocentricity Index: 3r+ (2)/R An index computed as 3 times the number of reflections plus pairs divided by R

es: Experienced Stimulation, the sum of FM+m and SumC’+SumT+SumY+SumV

Ex: Content code for reference to an explosion, including fireworks

F: A determinant reflecting the use of only pure form in generating the response

FABCOM: Fabulized Combination, Level one, a Special Score reflecting the use of an implausible relationship or transparency

FAB2: Fabulized Combination, Level two, a Special Score reflecting the use of an impossible relationship

FC: A determinant reflecting the emphasis of form over color in generating the response

FC’: A determinant reflecting the emphasis of form over achromatic color in generating the response

FD: Form Dimension, a determinant reflecting the use of dimensionality based on the contours of the blot in generating the response

Fd: Content code for reference to food that would be consumed by either animals or humans

Fi: Content code for reference to fire or smoke

FM: Animal movement, a determinant reflecting kinesthesis or activity in a response with animal content

FM+m: The addition of all Animal movement (FM) and all inanimate, inorganic, or insensate movement

‎(m)‎

Form Quality: Reflects the goodness of fit, the extent to which a response fits the portion of the blot used

‎(+,o,u,-)‎

FQx– : All the Form Quality minus in a record

FQxNone: All the Form Quality none in a record

FQxo: All the Form Quality ordinary in a record

FQx+: All the Form Quality ordinary-elaborated in a record

FQxu: All the Form Quality unusual in a record

Fr: A determinant indicating the emphasis of form over reflection in generating the response

Fr+rF: The addition sum of all reflection responses

FT: A determinant reflecting the emphasis of form over texture in generating the response

FV: A determinant reflecting the emphasis of form over dimensionality based on shading in generating the response

FY: A determinant reflecting the emphasis of form over shading in generating the response

Ge: Content code for reference to a map

Good HR (GHR): Human content answers not characterized by minus Form Quality, significant cognitive slippage, Hd, AN content, ‎MOR or AG Special Scores

H: Content code for reference to a real, whole human figure

‎(H): Content code for reference to mythological or fictional whole human figure

Hd: Content code for reference to a real human detail

‎(Hd): Content code for reference to mythological or fictional human detail

H+ (H) + Hd + (Hd): All human contents to include whole real and mythological as well as details of real and mythological humans

Hh: Content code for reference to household items

HVI Total: The total of all variables that constitute the Hypervigilance Index

Hx: Content code for reference to human experience including emotions and sensory experiences

INCOM: Incongruous Combination Level one, a Special Score reflecting the attribution of some aspect or activity to a response that is ‎out of keeping with that response

INC2: Incongruous Combination Level two, a Special Score reflecting the attribution of some aspect or activity to a response that is ‎out of keeping with that response which is bizarre, illogical, or beyond reality

Idiographic: Content that is not captured by other Content categories

Inquiry: A procedure following the response phase to aid in the accurate coding of responses

Intellect Intellectualization Index: (2AB+Art+Ay) ‎

Isolate/R Isolation Index: (Bt + 2Cl+Ge+Ls+2Na/R)‎

Lambda: A ratio of pure Form responses to all other responses (F/R-F)‎

Location: That part of the coding that reflects where the percept is in the blot

Ls: Content code for reference to any landscape concept

Lvl 2 Sp Sc: Level 2 Special Score; assigned to Special Scores that contain a bizarre or severe quality

m: Inanimate movement, a determinant reflecting kinesthesis or activity in a response involving inanimate, insensate, or inorganic ‎concepts

M: Human movement, a determinant reflecting kinesthesis or activity involving humans, or inanimate concepts or animals engaged in ‎nonspecies specific movement

Ma: Human Movement that is active

MOR: Morbid Content, a Special Score used to reflect a response that contains an object that is broken, destroyed, or damaged or to ‎indicate the presence of dysphoric affect

Mp: Human Movement that is passive

MQNone: All the Form Quality None for Human Movement (M) in a record

MQo: All the Form Quality ordinary for Human Movement (M) in a record

MQ+: All the Form Quality ordinary-elaborated for Human Movement (M) in a record

MQu: All the Form Quality unusual for Human Movement (M) in a record

MQ –: All the Form Quality minus for Human Movement (M) in a record

Na: Content code for reference to any concept from the natural environment

p: (passive) Superscript for movement determinants denoting a lower level of behavioral output than a (active); benchmarks of ‎‎“talking” and “looking” are passive

Pair (2): The use of the symmetry of the blot to produce two identical percepts in a response

PER: A Special Score that reflects the use of personal knowledge or experience to justify a response; often PER contains “I,” “me,” or ‎‎“my”‎

Poor HR (PHR): Human content answers characterized by minus Form Quality, significant cognitive slippage,‎

Hd, AN content, MOR, or AG Special Scores

Popular (P): A response that occurs with unusually high frequency, at least once in every three protocols

PSV: Perseveration, a Special Score reflecting a form of cognitive rigidity that manifests in providing two or more very similar ‎responses to the same blot or referring to previously articulated material and indicating that it is the same as previously seen

PTI Total: The total of all variables that constitute the Perceptual-Thinking Index

PureF%: The total number of F determinants divided by R

R: An answer; a response to any location in the blot

rF: A determinant indicating the emphasis of reflection over form in generating the response

S: Location code indicating a response that includes white space

Sc: Content code for reference to a product of science, science fiction, or industry

Special Scores: That part of the coding that addresses special aspects of how the response is worded

Sum C’: All of the achromatic determinants in a record

Sum Color: All of the Color determinants in a record

Sum Shading: All of the Shading (Sum Y, Sum V and Sum T) and Achromatic (Sum C’) determinants in a record

Sum 6 Sp Sc: All of the Cognitive Special Scores in a record (DV, INC, DR, FAB, A LOG, and CONTAM)‎

Sum T: All of the Texture determinants in a record

Sum V: All of the Vista determinants in a record

Sum Y: All of the Diffuse Shading determinants in a record

Sx: Content code for reference to concepts of a sexual nature including body parts and sexual activity

T: A determinant reflecting the use of only texture in generating the response

TF: A determinant reflecting the emphasis of texture based on shading over form in generating the response

V: A determinant reflecting the use of only dimensionality based on shading in generating the response

VF: A determinant reflecting the emphasis of dimensionality based on shading over form in generating the response

W: Location code indicating response uses the entire card

WDA%: A Form Quality calculation derived by adding FQ+, FQo, and FQu for W and D locations divided by the total number of W + ‎D responses

WD–: Form Quality Minus for all W and all D responses

WD None: Form Quality None for all W and all D responses

WD o: Form Quality Ordinary for all W and all D responses

WD+: Form Quality Plus for all W and all D responses

WD u: Form Quality Unusual for all W and all D responses

WSumC: Weighted Sum Color derived by (0.5)*FC + (1.0)*CF + (1.5)*C

WSum6: Weighted Sum6, the weighted sum of the six Cognitive Special Scores (DV, INC, DR, FAB, ALOG, and CONTAM)‎

XA%: A Form Quality calculation derived by adding FQ+, FQo, and FQu divided by R

X+%: A Form Quality calculation derived by adding FQ+ and FQo divided by R

X–%: A Form Quality calculation derived by dividing the total number of FQ–divided by R

Xu%: A Form Quality calculation derived by dividing the total number of FQu by R

Xy: Content code for reference to an X-ray

Y: A determinant reflecting the use of only shading in generating the response

YF: A determinant reflecting the emphasis of shading over form in generating the response

Zd: ZSum—Zest

Zf: The total number of Z scores in a record
